# Boron Nitride Nanotubes Versus Carbon Nanotubes: A Thermal Stability and Oxidation Behavior Study

**DOI:** 10.3390/nano10122435

**Published:** 2020-12-05

**Authors:** Nikolaos Kostoglou, Christos Tampaxis, Georgia Charalambopoulou, Georgios Constantinides, Vladislav Ryzhkov, Charalabos Doumanidis, Branko Matovic, Christian Mitterer, Claus Rebholz

**Affiliations:** 1 Department of Materials Science, Montanuniversität Leoben, 8700 Leoben, Austria; christian.mitterer@unileoben.ac.at (C.M.); claus@ucy.ac.cy (C.R.); 2 National Center for Scientific Research Demokritos, 15341 Athens, Greece; c.tampaxis@inn.demokritos.gr (C.T.); gchar@ipta.demokritos.gr (G.C.); 3 Department of Mechanical and Materials Science and Engineering, Cyprus University of Technology, 3036 Lemesos, Cyprus; g.constantinides@cut.ac.cy; 4 Research School of High-Energy Physics, Tomsk Polytechnic University, 634050 Tomsk, Russia; ryzhkov@tpu.ru; 5 College of Engineering and Computer Science, VinUniversity, Hanoi 100000, Vietnam; hdoumani@gmail.com; 6 Vinča Institute of Nuclear Sciences, University of Belgrade, 11000 Belgrade, Serbia; mato@vin.bg.ac.rs; 7 Department of Mechanical and Manufacturing Engineering, University of Cyprus, 1678 Nicosia, Cyprus

**Keywords:** boron nitride nanotubes, carbon nanotubes, purity, thermal stability, oxidation resistance

## Abstract

Nanotubes made of boron nitride (BN) and carbon have attracted considerable attention within the literature due to their unique mechanical, electrical and thermal properties. In this work, BN and carbon nanotubes, exhibiting high purity (>99%) and similar surface areas (~200 m^2^/g), were systematically investigated for their thermal stability and oxidation behavior by combining thermal gravimetric analysis and differential scanning calorimetry methods at temperatures of up to ~1300 °C under a synthetic air flow environment. The BN nanotubes showed a good resistance to oxidation up to ~900 °C and fully transformed to boron oxide up to ~1100 °C, while the carbon nanotubes were stable up to ~450 °C and almost completely combusted up to ~800 °C. The different oxidation mechanisms are attributed to the different chemical nature of the two types of nanotubes.

## 1. Introduction

Nanostructured materials have been a “hot” research topic in recent years since improved properties are expected compared to their bulk phase counterparts [1,2]. In this respect, one-dimensional tubular structures with nano-sized diameters, widely known as nanotubes, have attracted significant attention over the past three decades. Carbon nanotubes (CNTs) and boron nitride nanotubes (BNNTs), the most well-known nanotubular representatives, share the same crystal structure (i.e., sp^2^-bonded atoms forming hexagonal rings), even though they are composed of different elements [3,4]. However, the C-C bond is purely covalent while the B-N bond is partially ionic due to the difference in electronegativity between B and N elements [5]. Although CNTs and BNNTs exhibit similar properties (e.g., tensile strength, Young’s modulus, thermal conductivity), they also demonstrate profound differences (e.g., CNTs are metallic or semiconducting while BNNTs are electrically insulating) [6]. Both types of nanotubes have been considered for a series of emerging applications, even for controlled and targeted drug delivery [7,8]

**Figure 1:**
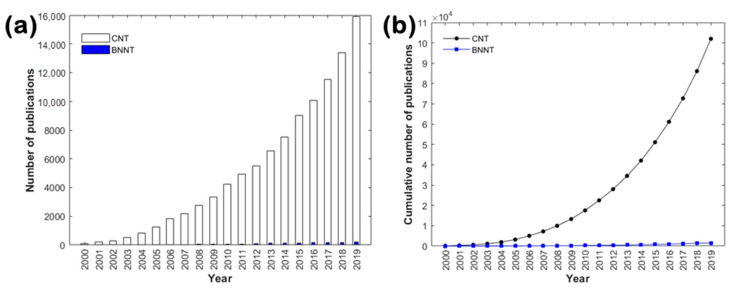
Search results in Scopus (September 2020) for (**a**) the annual number of peer-reviewed publications on BNNTs and CNTs (the solid bars for BNNTs can be barely noticed) and (**b**) the cumulative number of peer-reviewed publications between the years 2000 and 2019.

Despite their remarkable properties, BNNTs have received far less attention than CNTs within the literature. More specifically, a detailed search on the Scopus database for peer-reviewed publications on BNNTs and CNTs between the years 2000 and 2019 revealed a distinct difference in their total number (see Figure 1). Characteristically, only ~200 papers related to BNNTs were published in 2019 in contrast to ~15,900 papers referring to CNTs (i.e., ratio of ~1:80), as seen in Figure 1a. Furthermore, the cumulative number of publications on BNNTs hardly reached 1600 (in the period 2000–2019), while the respective ones on CNTs exceeded 100,000 (see Figure 1b). Such a characteristic difference can be attributed to the greater difficulty in the production of BNNTs and by consequence to the limited availability of BNNTs over the time [9].

A key property of BNNTs, also substantially different compared to CNTs, is related to their superior high-temperature resistance, even in atmospheric conditions, which opens new pathways for emerging applications (e.g., aerospace industry) where robust materials are required to withstand extreme conditions [10]. In this context, it has been suggested that crystallinity, particle shape and size, specific surface area (SSA), and purity play a significant role in the thermal stability and oxidation behavior of BN materials [11,12,13]. More specifically, crystalline BN materials with large particle size or small SSA seem to provide less reactive sites favorable for oxidation. We have previously investigated highly crystalline and pure (>98%) BN nanoplatelets with a low SSA (~23 m^2^/g) [14], as well as porous and highly pure (>99%) BN nanoplatelets with a larger SSA (~213 m^2^/g) [13] and reported oxidation resistance temperatures of up to ~1000 °C and ~800 °C, respectively. Taking into consideration that most of the previous studies found in the literature, focusing on the comparison of laboratory-synthesized BNNTs and CNTs, have shown a lack of high-purity nanotubes (i.e., a significant amount of catalytic remnants can influence the oxidation behavior) and/or have not considered the SSA values of these materials (i.e., lack of reports on gas sorption data), the comparison of the high-temperature properties of high-purity BNNTs and CNTs with similar SSA values is clearly of fundamental interest.

The current study presents a systematic investigation and comparison of the high-temperature properties of two isostructural materials (BNNTs and CNTs) of high purity that demonstrate different chemistry but similar crystallinity and SSA. The respective oxidation mechanisms are highlighted for each case and the influence of the chemical nature is discussed. Properties related to morphology, microstructure, surface chemistry, and porosity were deduced using scanning electron microscopy (SEM), transmission electron microscopy (TEM), X-ray diffraction (XRD), Fourier-transform infrared spectroscopy (FT-IR), and N_2_ adsorption/desorption measurements at −196 °C. The thermal stability and oxidation behavior were examined by combining thermal gravimetric analysis (TGA) and differential scanning calorimetry (DSC) under synthetic air-flow conditions.

## 2. Materials and Methods

Multi-walled BNNTs were obtained from BNNT LLC Company (Newport News, VA, USA) and were produced by a high temperature-pressure method, also known as pressurized vapor condenser method [10]. Multi-walled CNTs were acquired from Chengdu Organic Chemicals Company Ltd. Chinese Academy of Sciences (Chengdu, China) and were synthesized by chemical vapor deposition via decomposition of natural gas over a Ni-based catalyst followed by chemical purification using a mixture of potassium permanganate and sulfuric acid.

SEM images were collected by a FEI Quanta 200 microscope (Hillsboro, OR, USA) using a 20 kV acceleration voltage and a working distance of 10 mm. Prior to SEM analysis, the samples were sputter-coated with Au with the aim to improve their conductivity and avoid potential charging effects during imaging. For an alternative and rapid assessment of the surface area to volume ratio, fractal dimension analysis of the SEM images was performed by box-counting algorithms of four different measures (capacity, information, correlation, and probability) using the FDim image processing software [15]. TEM micrographs were recorded by a Philips CM-20 microscope (Eindhoven, Netherlands) with high-resolution capabilities equipped with a LaB_6_ filament using a 200 kV acceleration voltage. The samples were ultra-sonicated in ethanol and were placed onto holey carbon-only support films mounted on copper grids. A statistical analysis of the outer and inner diameters for both types of nanotubes was carried out by measuring 150 nanotubes (for each case) from low and high-magnification TEM images.

X-ray diffractograms were collected by a Bruker-AXS D8 Advance diffractometer (Karlsruhe, Germany) equipped with CuKα radiation (λ ~ 0.154 nm) at 40 kV voltage and 40 mA current. The XRD measurements were performed using a continuous scan speed mode in the diffraction angle range 2θ = 10–60°, a 0.01° step width and a 0.5 °/min scan speed in Bragg–Brentano geometry. The interlayer distances were calculated from the (002) reflections using Bragg’s law, while the crystallite thicknesses and crystallite lateral sizes were estimated from the (100)/(101) reflections using Scherrer formula. FT-IR spectra were recorded by a JASCO-4100 spectrometer (Shangai, China) in the mid-infrared region between 4400 and 400 cm^−1^. Samples of ~1 mg were prepared in a pellet form by using potassium bromide.

N_2_ adsorption/desorption isotherms were collected at −196 °C using a Quantachrome Autosorb-1-MP gas sorption analyzer (Boynton Beach, FL, USA) and ultra-pure (99.999%) N_2_. Before the measurements, samples of ~50 mg were degassed under high vacuum (10^−6^ mbar) at 250 °C for 24 h. The Brunauer–Emmet–Teller (BET) area values were calculated following the pertinent consistency criteria (ISO 9277:2010).

TGA and DSC curves were simultaneously recorded by a SETARAM SETSYS Evolution-18 thermal analyzer (Caluire, France) in the temperature range 25–1300 °C using a 10 °C/min heating rate and a 16 mL/min synthetic air flow. Samples of ~10 mg were placed in alumina crucibles, while buoyancy effects were taken into consideration by performing a blank measurement. Prior to thermal analysis, purging was applied to remove residual contamination from inside the furnace.

## 3. Results and Discussion

### 3.1. Surface Morphology

**Figure 2:**
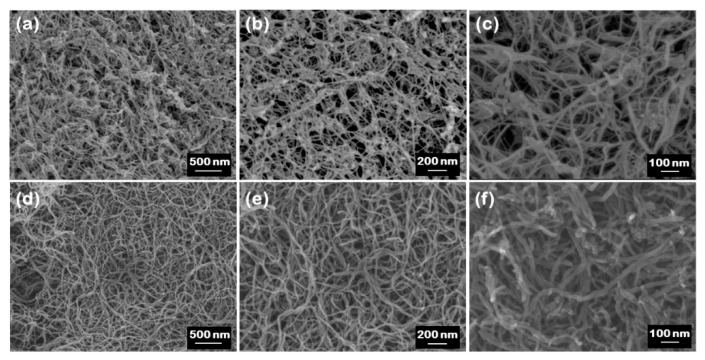
SEM images at different magnifications for (**a**–**c**) BNNTs and (**d**–**f**) CNTs.

**Figure 3:**
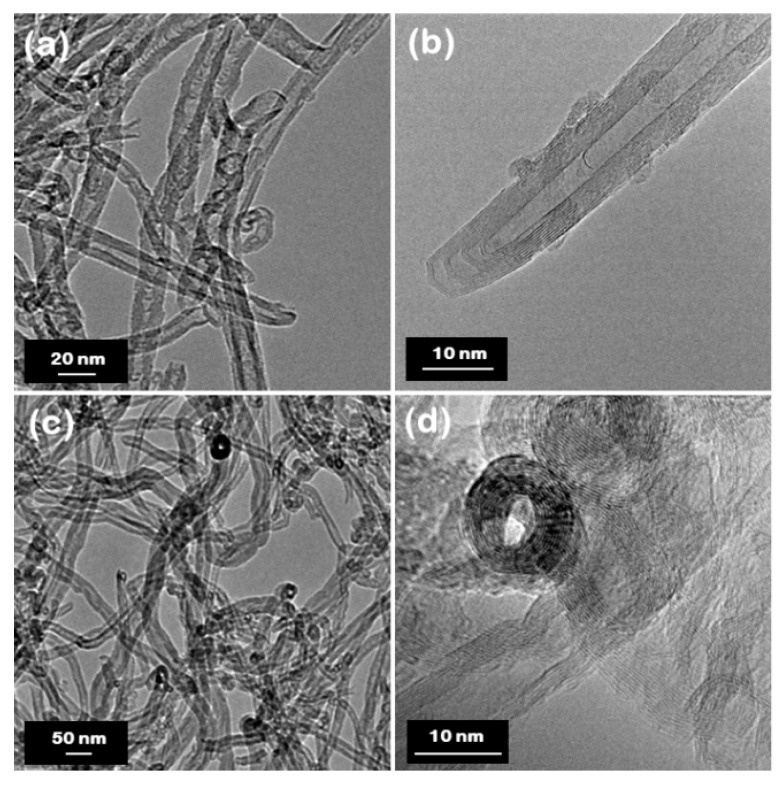
TEM images at different magnifications for (**a**,**b**) BNNTs and (**c**,**d**) CNTs.

**Figure 4:**
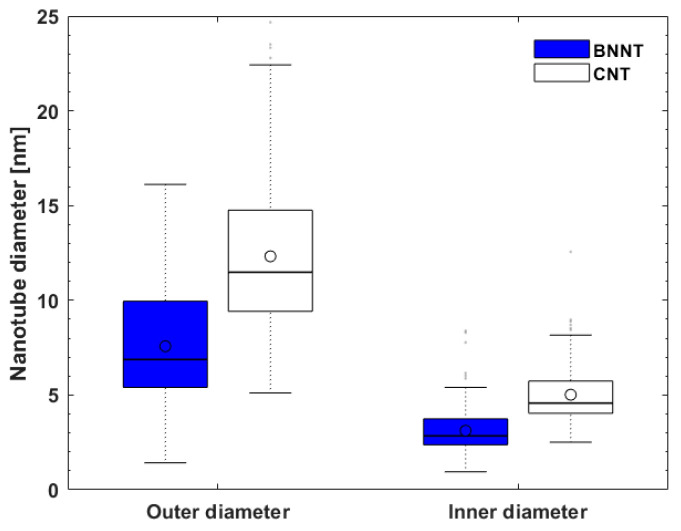
Box plots for the outer and inner nanotube diameters of the BNNTs and CNTs as measured by low- and high-magnification TEM images; the circle marker and the bold line correspond to the mean and median values, respectively.

Figure 2a–f display SEM images at different magnifications for the BNNTs and CNTs. The BNNTs consist of nanotube bundles held together in a ‘web-like’ structure (see Figure 2a–c), while the CNTs exhibit a ‘spaghetti-like’ morphology (see Figure 2d–f). Both materials present a typical multi-walled nanotubular structure, as evidenced from the high-resolution TEM images (see Figure 3). Open-ended nanotubes can be mostly observed in the case of CNTs (see Figure 3c,d), which is most likely attributed to the chemical purification process that followed their production. The BNNTs seem to exhibit a slightly smaller outer and inner diameter (7.6 ± 2.8 nm and 3.1 ± 1.3 nm, respectively) compared to the CNTs (12.3 ± 4.3 nm and 5.0 ± 1.6 nm, respectively), as determined by low- and high-magnification TEM images. More information about the statistical analysis in the nanotube diameters can be found in the box plots of Figure 4. The average values of the fractal dimension parameters, measured with four different algorithms, showed only a 4% difference between the two materials (see Table 1), thus suggesting comparable surface area-to-volume ratios. 

### 3.2. Microstructure and Surface Chemistry

**Figure 5:**
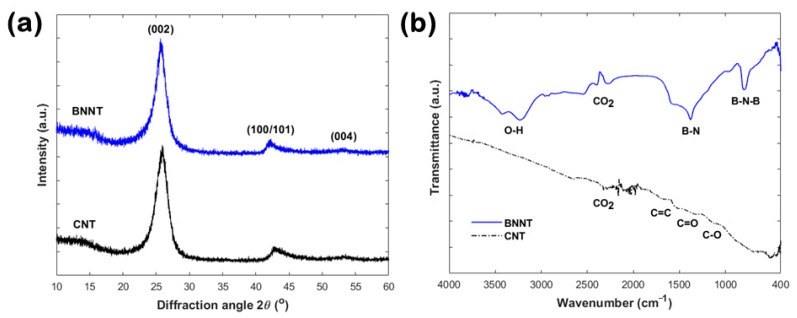
(**a**) X-ray diffractograms and (**b**) FT-IR spectra for the BNNTs and CNTs.

The XRD and FT-IR analysis for the BNNTs and CNTs are presented in Figure 5. The X-ray diffractograms (see Figure 5a) indicated very similar structural characteristics for the two materials. The BNNTs pattern shows features related to the hexagonal BN structure (JCPDS card no. 34-0421), including the (002), (100)/(101), and (004) reflections at 2θ ~25.6°, ~42.3°, and ~53.1°, respectively. Similarly, the graphitic (002) reflection at 2θ ~25.9° is dominating the CNTs pattern (JCPDS card no. 75-1621), followed by the less-pronounced (100)/(101) and (004) reflections at ~42.8° and ~53.5°, respectively. The CNTs pattern is slightly shifted to higher 2θ angles, thus indicating slightly narrower interlayer spacing. The interlayer distances for BNNTs and CNTs were estimated at ~0.348 and ~0.344 nm, respectively, which are larger than those of hexagonal BN (0.330–0.333 nm) and graphite (0.333–0.335 nm) [16]. The average crystallite thicknesses and crystallite lateral sizes were estimated around 3 nm and 12 nm, respectively, for both materials. No other peaks related to impurities were detected in both cases, which confirms the high purity of these materials in terms of BN and carbon, respectively. Similar XRD patterns for high-purity BNNTs and CNTs have been reported in recent studies [17,18].

The FT-IR spectra (see Figure 5b) revealed distinctively different surface chemistries for the two materials. The BNNTs exhibit characteristic bands at ~795 and ~1377 cm^−1^, attributed to the out-of-plane B–N–B bending and the in-plane B–N stretching vibration modes, respectively [19]. Less-intensive and broader bands around ~2365 and ~3400 cm^−1^ are due to atmospheric CO_2_ and adsorbed H_2_O, respectively. In contrast, the CNTs spectrum is almost featureless with a low signal-to-noise ratio due to intense absorption of infrared radiation from the carbon. Weak and broad bands shown in the 1000–1700 cm^−1^ region are probably due to the C–O/C=O vibrations of oxygen-based functionalities (e.g., carboxyls) and the C=C stretching vibration mode of sp^2^ carbon [20]. The CO_2_ band is also visible in this case. The carbon nature of the CNTs was also confirmed by Raman spectroscopy, where typical disorder-induced and graphitic bands were observed (see Figure S1 in Supplementary Materials).

### 3.3. Porosity and Surface Area

**Figure 6:**
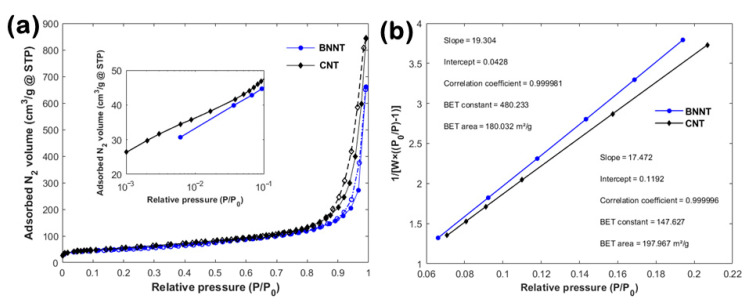
(**a**) N_2_ adsorption (solid symbols) and desorption (open symbols) isotherms recorded at −196 °C for the BNNTs and CNTs; the inset shows the N_2_ adsorption behavior at the lower relative pressures (*P/P*_0_ < 10^−1^) using a logarithmic scale and (**b**) linear multi-point BET plots calculated at the relative pressure range 0.06 < *P/P*_0_ < 0.22.

Both materials demonstrated a comparable N_2_ adsorption/desorption behavior at −196 °C (see Figure 6a), thus suggesting a similar pore structure. The main difference is the slightly increased adsorbed N_2_ volume of the CNTs both at lower (i.e., *P/P*_0_ < 0.1; see Figure 6a inset) and higher (i.e., *P/P*_0_ ~ 0.99) relative pressures. The small hysteresis loop formed between the adsorption and the desorption curves at *P/P*_0_ > 0.85 is attributed to capillary condensation of N_2_ in mesopores (i.e., pore widths 2–50 nm) [21]. The sudden rise of the adsorption curve at *P/P*_0_ ~ 0.99 and the lack of a saturation “plateau” describe condensation of N_2_ in macropores (i.e., pore widths larger than 50 nm) or N_2_ adsorption onto external surfaces [22]. The calculated BET areas (see Figure 6b) showed only a ~10% difference, i.e., ~180 and ~198 m^2^/g for BNNTs and CNTs, respectively.

### 3.4. Thermal Stability and Oxidation Behavior

**Figure 7:**
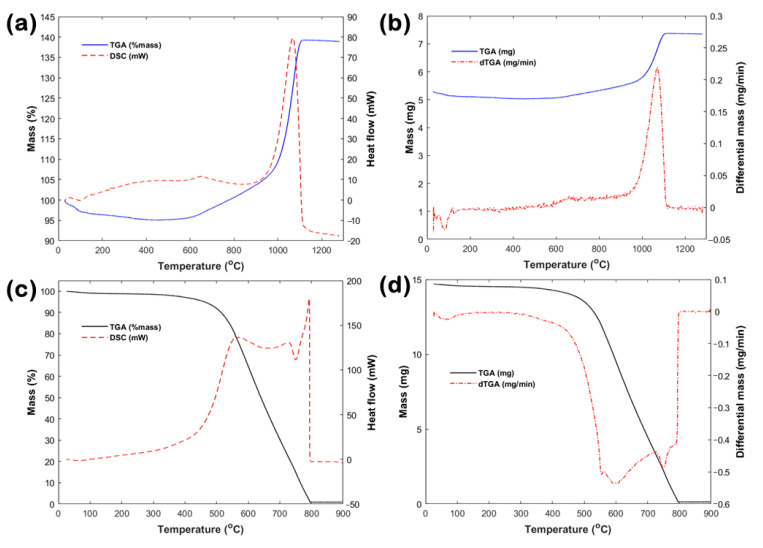
(**a**) Combined TGA/DSC and TGA/dTGA curves in synthetic air for (**a**,**b**) BNNTs and (**c**,**d**) CNTs; dTGA stands for differential TGA (temporal mass change).

TGA and DSC curves under synthetic air (Figure 7) suggested completely different oxidation behaviors for the BNNTs and CNTs. For the BNNTs (Figure 7a), the initial mass decreased by ~4% up to ~150 °C due to desorption of H_2_O, CO_2_, and other physisorbed surface contaminations [23]. This can be identified by the weak endothermic DSC peak shown below 150 °C and is much clearer in the differential TGA (dTGA) curve (Figure 7b). Between ~150 and 600 °C, the BNNTs mass remained stable (only decreased by another ~1%) with no pronounced DSC signals, thus indicating the absence of any chemical reactions or phase transformations up to ~600 °C. The decomposition initiated at temperatures beyond 600 °C and the subsequent oxidation was completed at ~1100 °C. The boron reacts with oxygen (O_2_) from the synthetic air and produces boron trioxide (B_2_O_3_), while the nitrogen is released as a molecular gas (N_2_), based on the chemical reaction 4BN + 3O_2_ → 2B_2_O_3_ + 2N_2_. Nitric oxide (NO_x_) gases might be also released during oxidation, as shown previously [24].

In more detail, the BN oxidation can be distinguished in two regions: (a) initial oxidation between ~600 and 900 °C and (b) active oxidation between ~900 and 1100 °C. For (a), a mass gain of ~10% was observed between ~600 and 900 °C, most probably due to oxidation of external/distorted nanotube layers and B_2_O_3_ formation [25]. The DSC and dTGA curves indicated only a broad peak centered around 650 °C. For (b), the TGA curve showed a steeper rise between ~900 and 1100 °C and finally reached a saturation point (mass increased further by ~33%), most likely attributed to oxidation of inner nanotube layers and complete transformation to B_2_O_3_ [13]. The strong exothermic DSC peak as well as the fast reaction rate of the dTGA curve (see Figure 7b) in the same range further confirmed the above observations. The oxidation did not proceed beyond 1100 °C and the excess oxidized mass remained stable up to ~1300 °C. It should be noted that the total mass increased by ~43% between ~600 and 1100 °C, which agrees well with the theoretical maximum mass gain of 40.3% by complete BN oxidation into B_2_O_3_.

For the CNTs (Figure 7c), the initial mass decreased by ~4% up to ~450 °C. The mass loss below 200 °C is attributed to removal of physisorbed species [26], as indicated by the broad ‘hump’ in the dTGA curve (see Figure 7d). The active oxidation initiated at temperatures beyond 450 °C and an almost complete ‘burnout’ was achieved at ~800 °C (i.e., drastic mass loss by ~95%). The carbon reacts with O_2_ from the synthetic air and gaseous CO_2_ is released. Three different oxidation profiles are identified from the exothermic DSC signals at ~550, ~750, and ~800 °C, respectively. Such distinctive oxidation behaviors are related to the diverse reactivity of inner and outer nanotube layers to thermal decomposition [20,26]. The DSC peaks agree well with the dTGA trend in the same temperature range (see Figure 7d). The minor residual mass (~1%), left upon combustion and up to ~900 °C, could be a synthesis remnant (e.g., metal catalyst) used to produce the CNTs.

To sum up, the main difference between the oxidation of BNNTs and CNTs lies in the form of their produced oxides. On the one side, B_2_O_3_ forms as a solid deposit right onto the oxidizing nanotube surface, thus retarding the oxidation process of the BNNTs. On the other side, CO_2_ is a volatile gaseous product, which hardly retards the oxidation process of the CNTs, while carbon oxidation to CO_2_ also generates defects/holes in the nanotube structure, thus further facilitating the combustion of the CNTs.

## 4. Conclusions

High-purity (>99%) BNNTs and CNTs, with comparable BET areas (~200 m^2^/g), were thermally analyzed up to 1300 °C in synthetic air. BNNTs withstood temperatures of up to ~900 °C prior to complete transformation to B_2_O_3_ at ~1100 °C, while CNTs remained stable only up to ~450 °C and almost fully ‘burned’ at 800 °C. The different oxidation profiles are explained by the different chemical nature of the nanotubes and the formation of different oxides during combustion.

## Figures and Tables

**Table 1:** Fractal dimension parameters deduced from SEM images (Figure 2).

Material	SEM Image	Capacity	Information	Correlation	Probability
BNNTs	Figure 2a	2.51	2.40	2.17	2.36
BNNTs	Figure 2b	2.53	2.40	2.16	2.30
BNNTs	Figure 2c	2.39	2.25	2.12	2.30
CNTs	Figure 2d	2.55	2.43	2.19	2.46
CNTs	Figure 2e	2.49	2.38	2.19	2.39
CNTs	Figure 2f	2.30	2.18	2.09	2.26

## Supplementary Materials

The following are available online at https://www.mdpi.com/2079-4991/10/12/2435/s1, Figure S1: Raman spectrum for the CNTs.
