# Supplementary material for: Boron Nitride Nanotubes Versus Carbon Nanotubes: A Thermal Stability and Oxidation Behavior Study
Source: Nanomaterials (Basel). 2020 Dec 5;10(12):2435. doi: 10.3390/nano10122435 (PMC7762177; doi:10.3390/nano10122435)
Supplement: Supplementary file 1 [file nanomaterials-10-02435-s001.pdf]

Supplementary Material:
Boron Nitride Nanotubes Versus Carbon Nanotubes: A Thermal Stability and Oxidation Behavior Study

The Raman spectrum for the CNTs (see Figure S1) was recorded by a Jobin-Yvon LABRAM confocal spectrometer equipped with a frequency-doubled Nd-YAG laser emitting at 532.2 nm. The laser beam was focused onto the sample by an Olympus BX 40 microscope fitted with a ×50 long-working distance objective lens at a power density of 0.1 mW/μm^2 and a spatial resolution of 1.5 cm^{-1}. The sharp peaks at ~1361 and ~1593 cm^{-1} correspond to the defect-activated D-band arising from breathing vibrations of carbon atoms with sp^3 hybridization and the graphitic G-band due to bond stretching of sp^2 hybridized carbon atoms in a 2-D hexagonal lattice, respectively. In the high-frequency region, the CNTs exhibit less-pronounced second order overtones, the 2D-band at ~2695 cm^{-1} and the 2D′-band at ~3242 cm^{-1}, as well as the combination of the D+D′ defect activated mode at ~2941 cm^{-1}.

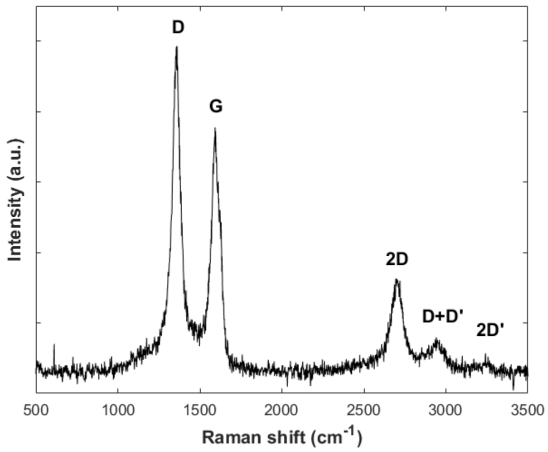

Figure S1. Raman spectrum for the CNTs.
